# The use of segmented regression in analysing interrupted time series studies: an example in pre-hospital ambulance care

**DOI:** 10.1186/1748-5908-9-77

**Published:** 2014-06-19

**Authors:** Monica Taljaard, Joanne E McKenzie, Craig R Ramsay, Jeremy M Grimshaw

**Affiliations:** 1 Clinical Epidemiology Program, Ottawa Hospital Research Institute, Ottawa Hospital, 1053 Carling Avenue Admin Services Building, ASB 2-004, Civic Box 693, Ottawa, ON K1Y 4E9, Canada; 2 Department of Epidemiology and Community Medicine, University of Ottawa, Ottawa, ON, Canada; 3 School of Public Health and Preventive Medicine, Monash University, The Alfred Centre, 99 Commercial Road, Melbourne, VIC 3004, Australia; 4 Health Services Research Unit, University of Aberdeen, Foresterhill, Aberdeen AB25 2ZD, UK; 5 Clinical Epidemiology Program, Ottawa Hospital Research Institute, The Ottawa Hospital - General Campus, 501 Smyth Road, Box 711, Ottawa, ON K1H 8L6, Canada; 6 Department of Medicine, University of Ottawa, Ottawa, ON, Canada

**Keywords:** Interrupted time series design, Segmented regression analysis, Quality improvement collaborative

## Abstract

**Background:**

An interrupted time series design is a powerful quasi-experimental approach for evaluating effects of interventions introduced at a specific point in time. To utilize the strength of this design, a modification to standard regression analysis, such as segmented regression, is required. In segmented regression analysis, the change in intercept and/or slope from pre- to post-intervention is estimated and used to test causal hypotheses about the intervention. We illustrate segmented regression using data from a previously published study that evaluated the effectiveness of a collaborative intervention to improve quality in pre-hospital ambulance care for acute myocardial infarction (AMI) and stroke. In the original analysis, a standard regression model was used with time as a continuous variable. We contrast the results from this standard regression analysis with those from segmented regression analysis. We discuss the limitations of the former and advantages of the latter, as well as the challenges of using segmented regression in analysing complex quality improvement interventions.

**Findings:**

Based on the estimated change in intercept and slope from pre- to post-intervention using segmented regression, we found insufficient evidence of a statistically significant effect on quality of care for stroke, although potential clinically important effects for AMI cannot be ruled out.

**Conclusions:**

Segmented regression analysis is the recommended approach for analysing data from an interrupted time series study. Several modifications to the basic segmented regression analysis approach are available to deal with challenges arising in the evaluation of complex quality improvement interventions.

## Background

An Interrupted Time Series (ITS) study is a powerful quasi-experimental design for evaluating effects of interventions when random assignment is not feasible [1]. In an ITS study, a series of observations on the same outcome before and after the introduction of an intervention are used to test immediate and gradual effects of the intervention. A major strength of this design is its ability to distinguish the effect of the intervention from secular change, that is, change that would have happened even in the absence of the intervention. Estimating the intervention effect is done by comparing the trend in the outcome after the intervention to the existing trend in the pre-intervention period, and is achieved through modifications to the standard regression analysis. In a basic segmented regression analysis [2-4], the time period is divided into pre- and post-intervention segments, and separate intercepts and slopes are estimated in each segment. Statistical tests of changes in intercepts and slopes pre- to post-intervention are carried out. By making a few simple changes to the data set-up and model specification, segmented regression analysis can easily be implemented in standard statistical software packages. An additional adjustment is usually required to account for serial autocorrelation, which arises because observations taken over time are usually correlated. Technical details for data preparation, model specification, and adjustment for autocorrelation are presented elsewhere [2,4]. Several examples of the use of segmented regression analyses in studies of quality improvement interventions have been published [5-7].

## Illustration

To illustrate the segmented regression analysis approach, we analysed data from a previously published study [8] that used an ITS design to evaluate the effectiveness of a collaborative intervention to improve quality in pre-hospital ambulance care for acute myocardial infarction (AMI) and stroke at 11 publicly funded ambulance organizations in England. A series of weekly measurements (the percentage of patients with a pre-hospital diagnosis of AMI and stroke who received a defined care bundle) between January 2010 and February 2012 was used to measure the impact of the collaborative intervention. The six-month pre-intervention period was defined as January to June 2010. The authors used logistic regression analysis of the data at each site, with the outcome being delivery of the care bundle and the predictor being time, modeled in two ways: first as a continuous variable across the entire study period, and then as a dichotomous indicator representing pre- and post-intervention periods. The estimated odds ratios (ORs) from the models that specified time as a continuous variable were combined across sites using fixed effects meta-analysis. The authors concluded that, over all sites, the collaborative intervention led to statistically significant improvements in ambulance care for AMI (OR 1.04 per month, 95% Confidence Interval [CI] 1.04, 1.04) and stroke (OR 1.06, 95% CI 1.05, 1.07).

**Figure 1:**
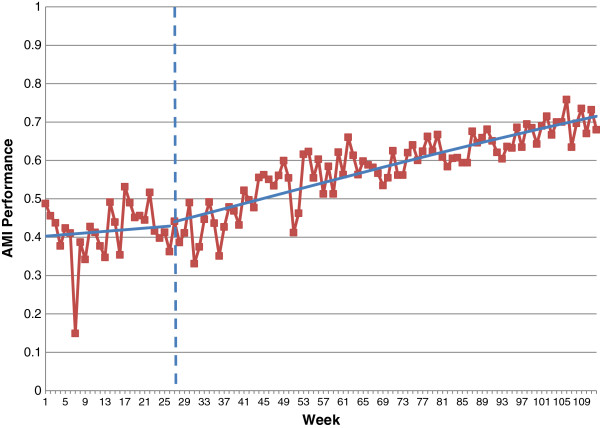
**Segmented logistic regression analysis of care bundle for AMI: all sites combined.**

**Figure 2:**
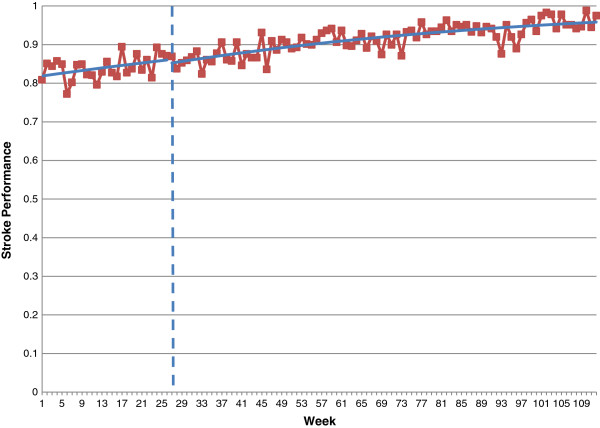
**Segmented logistic regression analysis of care bundle for stroke: all sites combined.**

We used the plots of weekly data aggregated across sites provided in the additional file [9] to re-analyse the data using segmented regression analysis. The details of our results are presented in Table 1 and displayed graphically in Figures 1 and 2. Before the intervention, the increase in AMI performance was OR = 1.02 per month; after the intervention, there was an additional increase of OR = 1.04 (95% CI 0.98 to 1.10) per month, which was not statistically significantly different from the pre-intervention trend (p = 0.20). Over the entire 86-week intervention period, the estimated increase in AMI performance is given by OR = 3.2 (a 220% relative increase); in the absence of the intervention, we would have expected a 45% increase (OR = 1.45). After accounting for the secular trend, the additional improvement associated with the intervention would, if it were real, likely be clinically important. For stroke, the pre-intervention increase in performance was OR = 1.05 per month; after the intervention, there was an additional effect of OR = 1.02 (95% CI 0.97 to 1.07) per month, which was not statistically significantly different from the pre-intervention trend (p = 0.52). Over the entire 86-week intervention period, the estimated increase in stroke performance is given by OR = 4 (that is, a 300% increase in odds). But even if the intervention had not been introduced, we would have expected a relative increase of 190% (OR = 2.9).

**Table 1:** **Segmented logistic regression analysis of care bundles for AMI and stroke**: **all sites combined**

**Parameter**	**Odds ratio (OR)**	**95% confidence interval for OR**	**p-value**
**AMI**			
Pre-intervention slope (secular trend, per month)	1.02	0.96 to 1.08	0.542
Change in intercept (immediate effect)	1.03	0.81 to 1.30	0.787
Change in slope (gradual effect, per month)	1.04	0.98 to 1.10	0.198
**Stroke**			
Pre-intervention slope (secular trend, per month)	1.05	1.00 to 1.10	0.038
Change in intercept (immediate effect)	0.93	0.75 to 1.14	0.465
Change in slope (gradual effect, per month)	1.02	0.97 to 1.07	0.517

## Discussion

A challenge in the use of the ITS design to evaluate complex quality improvement interventions is that the intervention may consist of several different components introduced at different times. For example, in this study, the authors’ preliminary investigations suggested that the two most effective interventions may be provider prompts and individualized feedback, while education and passive information dissemination did not appear to bring about change. One way to estimate the effects of different intervention components is to add multiple 'interruptions’ to the time series, but this requires a sufficient number of time points between interventions to allow their independent effects to be estimated [4]. In some studies, the intervention may need to be phased in or introduced gradually over a period of time, before being fully implemented. Thus, there may be a time lag from the initial introduction of an intervention to when its full effects can be observed. One way to allow for this in the segmented regression analysis is to fit a model with three segments, corresponding to the pre-implementation, implementation, and post-implementation periods. An alternative is to fit the model excluding the time points corresponding to the phase-in period [4,10]. Although the authors did not specify a phase-in period in their original analyses, we explored the effect of allowing for a phased introduction by censoring the first three months of observation after the start of the intervention. The results from this analysis are presented in Table 2, and our conclusions about the effect of the collaborative intervention remain unchanged.

**Table 2:** Segmented logistic regression analysis of care bundles for AMI and stroke: all sites combined, allowing for a ramp-up period of 12 weeks after introduction of the intervention

**Parameter**	**Odds ratio (OR)**	**95% confidence interval for OR**	**p-value**
**AMI**			
Pre-intervention slope (secular trend, per month)	1.02	0.97 to 1.07	0.362
Change in intercept (immediate effect)	1.16	0.93 to 1.44	0.199
Change in slope (gradual effect, per month)	1.02	0.97 to 1.08	0.346
**Stroke**			
Pre-intervention slope (secular trend, per month)	1.05	1.00 to 1.10	0.045
Change in intercept (immediate effect)	0.93	0.73 to 1.19	0.577
Change in slope (gradual effect, per month)	1.02	0.97 to 1.07	0.551

In ITS studies, it is not uncommon to have different participating sites contributing data. In our re-analyses of these data, we used a single time series of data aggregated across all sites. An analysis of aggregated data is likely to have less power than a multilevel logistic regression analysis of the time series from the individual sites. Gebski *et al*. [3] describe how to conduct segmented regression analysis when there are multiple sites and different intervention start times. One approach is to conduct separate segmented regression analyses at each site, and then estimate the overall effect by pooling the estimates of intervention effect across sites using inverse variance weights in a meta-analytical model [11]. Another approach is to fit a single model to the data from all sites and account for heterogeneity across sites by incorporating random effects for the sites.

Sensitivity analyses may be conducted to consider the effect of outliers on the results. Outliers may be censored from the analyses or modeled explicitly via dummy variables [2]. Alternatively, the effect of outliers may be reduced by using moving averages. Yet another approach is to combine data points prior to analysis, by using, for example, bi-weekly instead of weekly measurements. Although we did not explore these issues in our re-analyses, variability in outcomes over time can substantially affect power in an ITS study.

## Conclusion

In conclusion, our results demonstrate the importance of using segmented regression analysis in an ITS study. When a standard regression analysis is used with time modeled as a single continuous variable, an estimate is obtained for the slope over time, but it is impossible to distinguish the effect of the intervention from the underlying secular trend and to make causal claims about the effects of the intervention. Based on our re-analyses of these data, we conclude that the quality improvement collaborative resulted in no statistically significant improvements in the quality of AMI and stroke care, but that potential clinically important effects for AMI cannot be ruled out.

## Competing interests

The authors declare that they have no competing interests.

## Authors’ contributions

JMG conceived of this commentary, participated in discussions, and helped to draft the manuscript. MT participated in discussions, carried out the statistical analyses, and wrote the first draft of the manuscript. JEM participated in discussions and helped to draft the manuscript. CRR participated in discussions and helped to draft the manuscript. All authors read and approved the final manuscript.
